# Benzaldehyde thio­semicarbazone

**DOI:** 10.1107/S1600536808038270

**Published:** 2008-11-22

**Authors:** Lingqian Kong, Yan Qiao, Ji-Dong Zhang, Xiu-Ping Ju

**Affiliations:** a Dongchang College, Liaocheng University, Liaocheng 250059, People’s Republic of China

## Abstract

The title compound, C_8_H_9_N_3_S, contains two mol­ecules in the asymmetric unit. One mol­ecule is close to being planar (r.m.s. deviation from the mean plane = 0.06 Å for the non-H atoms), while the other exhibits a dihedral angle of 21.7 (1)° between the benzene ring and the mean plane of the thio­semicarbazone unit. Inter­molecular N—H⋯S hydrogen bonds link the mol­ecules into layers parallel to the (010) plane.

## Related literature

For background literature concerning aryl­hydrazone compounds, see: Beraldo & Gambino (2004 ▶); Bondock *et al.* (2007 ▶). For the related 2,4-dichloro­benzyl­idene compound, see: Jing *et al.* (2006 ▶).
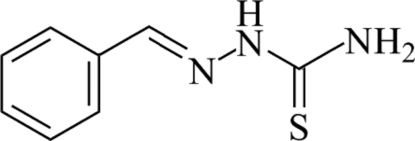


## Experimental

### 

#### Crystal data

C_8_H_9_N_3_S
*M* *_r_* = 179.24
Triclinic, 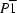

*a* = 5.8692 (13) Å
*b* = 12.513 (2) Å
*c* = 13.519 (2) Å
α = 112.735 (3)°
β = 95.384 (2)°
γ = 96.153 (2)°
*V* = 900.4 (3) Å^3^
*Z* = 4
Mo *K*α radiation
μ = 0.31 mm^−1^
*T* = 298 (2) K
0.24 × 0.13 × 0.10 mm

#### Data collection

Bruker SMART CCD area-detector diffractometer
Absorption correction: multi-scan (*SADABS*; Sheldrick, 1996 ▶) *T*_min_ = 0.930, *T*_max_ = 0.970
4740 measured reflections
3124 independent reflections
1846 reflections with *I* > 2σ(*I*)
*R*_int_ = 0.039

#### Refinement

*R*[*F* ^2^ > 2σ(*F* ^2^)] = 0.058
*wR*(*F* ^2^) = 0.162
*S* = 0.95
3124 reflections
217 parameters
H-atom parameters constrained
Δρ_max_ = 0.31 e Å^−3^
Δρ_min_ = −0.27 e Å^−3^

### 

Data collection: *SMART* (Siemens, 1996 ▶); cell refinement: *SAINT* (Siemens, 1996 ▶); data reduction: *SAINT*; program(s) used to solve structure: *SHELXS97* (Sheldrick, 2008 ▶); program(s) used to refine structure: *SHELXL97* (Sheldrick, 2008 ▶); molecular graphics: *SHELXTL* (Sheldrick, 2008 ▶); software used to prepare material for publication: *SHELXTL*.

## Supplementary Material

Crystal structure: contains datablocks I, global. DOI: 10.1107/S1600536808038270/bi2310sup1.cif

Structure factors: contains datablocks I. DOI: 10.1107/S1600536808038270/bi2310Isup2.hkl

Additional supplementary materials:  crystallographic information; 3D view; checkCIF report

## Figures and Tables

**Table 1:** Hydrogen-bond geometry (Å, °)

*D*—H⋯*A*	*D*—H	H⋯*A*	*D*⋯*A*	*D*—H⋯*A*
N2—H2⋯S1^i^	0.86	2.64	3.443 (3)	155
N3—H3*A*⋯S1^ii^	0.86	2.98	3.488 (3)	120
N5—H5⋯S1^ii^	0.86	2.61	3.441 (3)	162
N6—H6*B*⋯S2^iii^	0.86	2.51	3.368 (3)	173

Symmetry codes: (i) 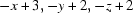; (ii) 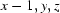; (iii) 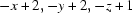.

**Fig. 1.**
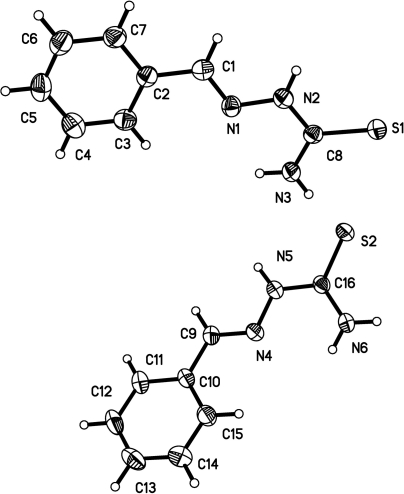
Two molecules in the asymmetric unit of the title compound with displacement ellipsoids shown at 30% probability for non-H atoms.

## supplementary crystallographic information

### Comment

Aryl-hydrazones, such as semicarbazones, thiosemicarbazones and guanyl hydrazones, often exhibit strong biological activity and are important compounds for drug design (Beraldo & Gambino, 2004), organocatalysis and the preparation of heterocyclic rings (Bondock *et al.*, 2007).

### Experimental

Benzaldehyde (0.3 mmol), thiosemicarbazide (0.3 mmol) and 10 ml water were mixed in a 50 ml flask. After stirring for 30 min at 373 K, the resulting mixture was recrystallized from ethanol, affording the title compound as colourless crystals. Elemental analysis: calculated C 53.61, H 5.06, N 23.44%; found: C 53.58, H 5.55, N 23.51%.

### Refinement

H atoms were placed in geometrically idealized positions (N—H = 0.86, C—H = 0.93 Å) and allowed to ride on their parent atoms with *U*_iso_(H) = 1.2*U*_eq_(C/N).

### Crystal data

**Table:**

C_8_H_9_N_3_S	*Z* = 4
*M**_r_* = 179.24	*F*_000_ = 376
Triclinic, *P*1	*D*_x_ = 1.322 Mg m^−^^3^
Hall symbol: -P 1	Mo *K*α radiation λ = 0.71073 Å
*a* = 5.8692 (13) Å	Cell parameters from 1310 reflections
*b* = 12.513 (2) Å	θ = 2.9–25.0º
*c* = 13.519 (2) Å	µ = 0.31 mm^−^^1^
α = 112.735 (3)º	*T* = 298 (2) K
β = 95.384 (2)º	Block, orange
γ = 96.153 (2)º	0.24 × 0.13 × 0.10 mm
*V* = 900.4 (3) Å^3^	

### Data collection

**Table:**

Bruker SMART CCD area-detector diffractometer	3124 independent reflections
Radiation source: fine-focus sealed tube	1846 reflections with *I* > 2σ(*I*)
Monochromator: graphite	*R*_int_ = 0.039
*T* = 298(2) K	θ_max_ = 25.0º
φ and ω scans	θ_min_ = 1.7º
Absorption correction: multi-scan(SADABS; Sheldrick, 1996)	*h* = −6→6
*T*_min_ = 0.930, *T*_max_ = 0.970	*k* = −12→14
4740 measured reflections	*l* = −16→14

### Refinement

**Table:**

Refinement on *F*^2^	Secondary atom site location: difference Fourier map
Least-squares matrix: full	Hydrogen site location: inferred from neighbouring sites
*R*[*F*^2^ > 2σ(*F*^2^)] = 0.058	H-atom parameters constrained
*wR*(*F*^2^) = 0.162	*w* = 1/[σ^2^(*F*_o_^2^) + (0.0856*P*)^2^] where *P* = (*F*_o_^2^ + 2*F*_c_^2^)/3
*S* = 0.95	(Δ/σ)_max_ < 0.001
3124 reflections	Δρ_max_ = 0.31 e Å^−^^3^
217 parameters	Δρ_min_ = −0.27 e Å^−^^3^
Primary atom site location: structure-invariant direct methods	Extinction correction: none

### Special details

**Table:**

Geometry. All e.s.d.'s (except the e.s.d. in the dihedral angle between two l.s. planes) are estimated using the full covariance matrix. The cell e.s.d.'s are taken into account individually in the estimation of e.s.d.'s in distances, angles and torsion angles; correlations between e.s.d.'s in cell parameters are only used when they are defined by crystal symmetry. An approximate (isotropic) treatment of cell e.s.d.'s is used for estimating e.s.d.'s involving l.s. planes.
Refinement. Refinement of *F*^2^ against ALL reflections. The weighted *R*-factor *wR* and goodness of fit *S* are based on *F*^2^, conventional *R*-factors *R* are based on *F*, with *F* set to zero for negative *F*^2^. The threshold expression of *F*^2^ > σ(*F*^2^) is used only for calculating *R*-factors(gt) *etc*. and is not relevant to the choice of reflections for refinement. *R*-factors based on *F*^2^ are statistically about twice as large as those based on *F*, and *R*- factors based on ALL data will be even larger.

### Fractional atomic coordinates and isotropic or equivalent isotropic displacement parameters (Å^2^)

**Table:**

	*x*	*y*	*z*	*U*_iso_*/*U*_eq_	
N1	0.9674 (5)	0.8260 (3)	0.9684 (2)	0.0484 (8)	
N2	1.1667 (5)	0.8822 (3)	0.9512 (2)	0.0504 (8)	
H2	1.2934	0.8999	0.9956	0.061*	
N3	0.9536 (5)	0.8863 (3)	0.8034 (2)	0.0550 (9)	
H3A	0.8331	0.8563	0.8208	0.066*	
H3B	0.9414	0.9020	0.7467	0.066*	
N4	0.3374 (5)	0.7659 (3)	0.4823 (2)	0.0486 (8)	
N5	0.4649 (5)	0.8653 (3)	0.5625 (2)	0.0518 (8)	
H5	0.4200	0.8950	0.6249	0.062*	
N6	0.7036 (6)	0.8737 (3)	0.4423 (3)	0.0612 (10)	
H6A	0.6092	0.8166	0.3930	0.073*	
H6B	0.8262	0.9031	0.4259	0.073*	
S1	1.40293 (17)	0.96409 (10)	0.83250 (8)	0.0561 (3)	
S2	0.83277 (19)	1.02721 (9)	0.64490 (8)	0.0610 (4)	
C1	0.9813 (7)	0.8063 (3)	1.0543 (3)	0.0500 (9)	
H1	1.1169	0.8341	1.1040	0.060*	
C2	0.7837 (7)	0.7398 (3)	1.0749 (3)	0.0493 (10)	
C3	0.5747 (7)	0.7039 (3)	1.0076 (3)	0.0569 (10)	
H3	0.5566	0.7229	0.9475	0.068*	
C4	0.3920 (8)	0.6403 (4)	1.0277 (4)	0.0660 (12)	
H4	0.2516	0.6167	0.9814	0.079*	
C5	0.4178 (9)	0.6118 (4)	1.1169 (4)	0.0700 (13)	
H5A	0.2950	0.5690	1.1309	0.084*	
C6	0.6255 (9)	0.6472 (4)	1.1845 (4)	0.0675 (12)	
H6	0.6431	0.6283	1.2447	0.081*	
C7	0.8081 (8)	0.7104 (3)	1.1640 (3)	0.0584 (11)	
H7	0.9486	0.7336	1.2100	0.070*	
C8	1.1574 (6)	0.9084 (3)	0.8637 (3)	0.0438 (9)	
C9	0.1484 (7)	0.7251 (3)	0.5034 (3)	0.0484 (9)	
H9	0.0969	0.7658	0.5687	0.058*	
C10	0.0129 (6)	0.6152 (3)	0.4263 (3)	0.0455 (9)	
C11	−0.2018 (7)	0.5775 (4)	0.4462 (4)	0.0607 (11)	
H11	−0.2622	0.6246	0.5065	0.073*	
C12	−0.3267 (8)	0.4700 (4)	0.3766 (4)	0.0695 (13)	
H12	−0.4716	0.4453	0.3898	0.083*	
C13	−0.2378 (9)	0.4002 (4)	0.2889 (4)	0.0736 (13)	
H13	−0.3205	0.3270	0.2435	0.088*	
C14	−0.0287 (9)	0.4368 (4)	0.2671 (4)	0.0730 (13)	
H14	0.0287	0.3893	0.2060	0.088*	
C15	0.0981 (7)	0.5436 (3)	0.3350 (3)	0.0576 (11)	
H15	0.2410	0.5680	0.3198	0.069*	
C16	0.6604 (6)	0.9162 (3)	0.5434 (3)	0.0443 (9)	

### Atomic displacement parameters (Å^2^)

**Table:**

	*U*^11^	*U*^22^	*U*^33^	*U*^12^	*U*^13^	*U*^23^
N1	0.0460 (19)	0.0479 (18)	0.0522 (19)	0.0088 (15)	0.0119 (15)	0.0195 (15)
N2	0.0434 (18)	0.056 (2)	0.0511 (19)	0.0056 (15)	0.0037 (15)	0.0225 (16)
N3	0.0415 (19)	0.072 (2)	0.0521 (19)	−0.0021 (16)	0.0018 (16)	0.0296 (17)
N4	0.0452 (19)	0.0479 (18)	0.0471 (18)	−0.0024 (15)	0.0008 (15)	0.0170 (15)
N5	0.048 (2)	0.0513 (19)	0.0488 (19)	−0.0080 (15)	0.0049 (15)	0.0170 (15)
N6	0.055 (2)	0.063 (2)	0.055 (2)	−0.0165 (17)	0.0107 (17)	0.0171 (17)
S1	0.0434 (6)	0.0700 (7)	0.0494 (6)	0.0008 (5)	0.0081 (5)	0.0196 (5)
S2	0.0595 (7)	0.0634 (7)	0.0504 (6)	−0.0189 (5)	−0.0037 (5)	0.0224 (5)
C1	0.054 (2)	0.046 (2)	0.049 (2)	0.0123 (19)	0.0080 (19)	0.0165 (18)
C2	0.060 (3)	0.039 (2)	0.047 (2)	0.0102 (19)	0.014 (2)	0.0133 (17)
C3	0.058 (3)	0.057 (3)	0.056 (2)	0.012 (2)	0.004 (2)	0.023 (2)
C4	0.054 (3)	0.060 (3)	0.082 (3)	0.013 (2)	0.011 (2)	0.024 (2)
C5	0.078 (3)	0.053 (3)	0.083 (3)	0.010 (2)	0.033 (3)	0.028 (2)
C6	0.088 (4)	0.058 (3)	0.060 (3)	0.011 (3)	0.021 (3)	0.026 (2)
C7	0.071 (3)	0.048 (2)	0.050 (2)	0.004 (2)	0.006 (2)	0.0163 (19)
C8	0.044 (2)	0.043 (2)	0.040 (2)	0.0094 (17)	0.0079 (18)	0.0112 (17)
C9	0.044 (2)	0.050 (2)	0.051 (2)	0.0031 (18)	0.0108 (18)	0.0215 (18)
C10	0.039 (2)	0.044 (2)	0.054 (2)	0.0005 (16)	0.0022 (17)	0.0224 (18)
C11	0.049 (2)	0.062 (3)	0.074 (3)	−0.001 (2)	0.013 (2)	0.032 (2)
C12	0.047 (3)	0.070 (3)	0.094 (4)	−0.012 (2)	−0.001 (2)	0.043 (3)
C13	0.078 (3)	0.048 (3)	0.081 (3)	−0.012 (2)	−0.015 (3)	0.022 (2)
C14	0.078 (3)	0.062 (3)	0.066 (3)	0.002 (3)	0.005 (3)	0.015 (2)
C15	0.052 (2)	0.053 (2)	0.063 (3)	0.000 (2)	0.007 (2)	0.020 (2)
C16	0.044 (2)	0.044 (2)	0.048 (2)	−0.0003 (17)	0.0004 (18)	0.0248 (18)

### Geometric parameters (Å, °)

**Table:**

N1—C1	1.274 (5)	C3—H3	0.930
N1—N2	1.385 (4)	C4—C5	1.384 (6)
N2—C8	1.342 (5)	C4—H4	0.930
N2—H2	0.860	C5—C6	1.371 (7)
N3—C8	1.320 (5)	C5—H5A	0.930
N3—H3A	0.860	C6—C7	1.377 (6)
N3—H3B	0.860	C6—H6	0.930
N4—C9	1.274 (5)	C7—H7	0.930
N4—N5	1.375 (4)	C9—C10	1.453 (5)
N5—C16	1.347 (4)	C9—H9	0.930
N5—H5	0.860	C10—C11	1.385 (5)
N6—C16	1.321 (4)	C10—C15	1.390 (5)
N6—H6A	0.860	C11—C12	1.383 (6)
N6—H6B	0.860	C11—H11	0.930
S1—C8	1.693 (4)	C12—C13	1.362 (6)
S2—C16	1.674 (4)	C12—H12	0.930
C1—C2	1.466 (5)	C13—C14	1.362 (6)
C1—H1	0.930	C13—H13	0.930
C2—C3	1.375 (5)	C14—C15	1.376 (6)
C2—C7	1.388 (5)	C14—H14	0.930
C3—C4	1.377 (6)	C15—H15	0.930
			
C1—N1—N2	116.5 (3)	C7—C6—H6	119.8
C8—N2—N1	118.4 (3)	C6—C7—C2	120.4 (4)
C8—N2—H2	120.8	C6—C7—H7	119.8
N1—N2—H2	120.8	C2—C7—H7	119.8
C8—N3—H3A	120.0	N3—C8—N2	117.6 (3)
C8—N3—H3B	120.0	N3—C8—S1	122.5 (3)
H3A—N3—H3B	120.0	N2—C8—S1	119.9 (3)
C9—N4—N5	117.1 (3)	N4—C9—C10	120.4 (4)
C16—N5—N4	120.0 (3)	N4—C9—H9	119.8
C16—N5—H5	120.0	C10—C9—H9	119.8
N4—N5—H5	120.0	C11—C10—C15	118.8 (4)
C16—N6—H6A	120.0	C11—C10—C9	119.6 (4)
C16—N6—H6B	120.0	C15—C10—C9	121.6 (3)
H6A—N6—H6B	120.0	C12—C11—C10	120.1 (4)
N1—C1—C2	120.0 (4)	C12—C11—H11	119.9
N1—C1—H1	120.0	C10—C11—H11	119.9
C2—C1—H1	120.0	C13—C12—C11	120.1 (4)
C3—C2—C7	118.8 (4)	C13—C12—H12	119.9
C3—C2—C1	121.9 (4)	C11—C12—H12	119.9
C7—C2—C1	119.3 (4)	C14—C13—C12	120.5 (4)
C2—C3—C4	121.0 (4)	C14—C13—H13	119.8
C2—C3—H3	119.5	C12—C13—H13	119.8
C4—C3—H3	119.5	C13—C14—C15	120.4 (5)
C3—C4—C5	119.8 (5)	C13—C14—H14	119.8
C3—C4—H4	120.1	C15—C14—H14	119.8
C5—C4—H4	120.1	C14—C15—C10	120.1 (4)
C6—C5—C4	119.7 (4)	C14—C15—H15	119.9
C6—C5—H5A	120.2	C10—C15—H15	119.9
C4—C5—H5A	120.2	N6—C16—N5	116.4 (3)
C5—C6—C7	120.4 (4)	N6—C16—S2	123.6 (3)
C5—C6—H6	119.8	N5—C16—S2	120.0 (3)
			
C1—N1—N2—C8	−177.7 (3)	N1—N2—C8—S1	−173.6 (2)
C9—N4—N5—C16	−176.7 (3)	N5—N4—C9—C10	−175.1 (3)
N2—N1—C1—C2	−175.6 (3)	N4—C9—C10—C11	−174.2 (4)
N1—C1—C2—C3	−4.5 (6)	N4—C9—C10—C15	8.9 (6)
N1—C1—C2—C7	174.5 (4)	C15—C10—C11—C12	0.6 (6)
C7—C2—C3—C4	0.2 (6)	C9—C10—C11—C12	−176.4 (4)
C1—C2—C3—C4	179.3 (4)	C10—C11—C12—C13	0.8 (7)
C2—C3—C4—C5	0.0 (6)	C11—C12—C13—C14	−1.9 (7)
C3—C4—C5—C6	0.0 (6)	C12—C13—C14—C15	1.7 (7)
C4—C5—C6—C7	−0.2 (7)	C13—C14—C15—C10	−0.2 (7)
C5—C6—C7—C2	0.4 (6)	C11—C10—C15—C14	−0.9 (6)
C3—C2—C7—C6	−0.4 (6)	C9—C10—C15—C14	176.0 (4)
C1—C2—C7—C6	−179.5 (3)	N4—N5—C16—N6	7.6 (5)
N1—N2—C8—N3	4.7 (5)	N4—N5—C16—S2	−172.6 (3)

### Hydrogen-bond geometry (Å, °)

**Table:**

*D*—H···*A*	*D*—H	H···*A*	*D*···*A*	*D*—H···*A*
N2—H2···S1^i^	0.86	2.64	3.443 (3)	155
N3—H3A···S1^ii^	0.86	2.98	3.488 (3)	120
N5—H5···S1^ii^	0.86	2.61	3.441 (3)	162
N6—H6B···S2^iii^	0.86	2.51	3.368 (3)	173

Symmetry codes: (i) −*x*+3, −*y*+2, −*z*+2; (ii) *x*−1, *y*, *z*; (iii) −*x*+2, −*y*+2, −*z*+1.
